# Effect of Di(2-ethylhexyl)phthalate on *Helicobacter pylori*-Induced Apoptosis in AGS Cells

**DOI:** 10.1155/2013/924769

**Published:** 2013-12-16

**Authors:** Chuang-Hao Lin, Chien-Yi Wu, Hwang-Shang Kou, Chiao-Yun Chen, Meng-Chuan Huang, Huang-Ming Hu, Meng-Chieh Wu, Chien-Yu Lu, Deng-Chyang Wu, Ming-Tsang Wu, Fu-Chen Kuo

**Affiliations:** ^1^Department of Physiology, College of Medicine, Kaohsiung Medical University, Kaohsiung City 807, Taiwan; ^2^Department of Pediatrics, E-Da Hospital, Kaohsiung City 824, Taiwan; ^3^School of Pharmacy, Kaohsiung Medical University, Kaohsiung City 807, Taiwan; ^4^Department of Medical Imaging, Kaohsiung Medical University Hospital, Kaohsiung City 807, Taiwan; ^5^Department of Nutritional Sciences, Kaohsiung Medical University Hospital, Kaohsiung, Taiwan; ^6^Division of Gastroenterology, Department of Internal Medicine, Kaohsiung Medical University Hospital, Kaohsiung City 807, Taiwan; ^7^Department of Medicine, Faculty of Medicine, College of Medicine, Kaohsiung Medical University, Kaohsiung City 807, Taiwan; ^8^Department of Internal Medicine, Kaohsiung Municipal Hsiao-Kang Hospital, Kaohsiung City 812, Taiwan; ^9^Cancer Center, Kaohsiung Medical University Hospital, Kaohsiung City 807, Taiwan; ^10^Graduate Institute of Public Health, Kaohsiung Medical University, Kaohsiung City 807, Taiwan; ^11^Department of Obstetrics and Gynecology, E-Da Hospital, Kaohsiung 824, Taiwan; ^12^School of Medicine, College of Medicine, I-Shou University, Kaohsiung City 840, Taiwan

## Abstract

Plastic products are wildly used in human life. Di(2-ethylhexyl)phthalate (DEHP) is an essential additive in plastic manufacturing and is used as plasticizer for many products including plastic food packaging. DEHP is a teratogenic compound and can cause potent reproductive toxicity. DEHP can also cause liver damage, peroxisome proliferation, and carcinogenesis. DEHP is also strongly associated with peptic ulcers and gastric cancer; however, the underlying effect and mechanism of DEHP on the gastrointestinal tract are not entirely clear. The oral infection route of *H. pylori* parallels the major ingestion route of DEHP into the human body. Therefore, we wanted to study the effect of DEHP and *H. pylori* exposure on the human gastric epithelial cell line, AGS (gastric adenocarcinoma). The viability of the AGS cell line was significantly lower in 80 **μ**M-DEHP and *H. pylori* (MOI = 100 : 1) coexposure than DEHP or *H. pylori* alone. DEHP and *H. pylori* coexposure also induced caspase-3 activation, and increased Bax/Bcl-2 ratio and DNA fragmentation in AGS cells. These results indicate that DEHP can enhance *H. pylori* cytotoxicity and induce gastric epithelial cell apoptosis. Therefore, it is possible that DEHP and *H. pylori* coexposure might enhance the disruption of the gastric mucosa integrity and potentially promote the pathogenesis of gastric carcinogenesis.

## 1. Introduction

Di(2-ethylhexyl)phthalate (DEHP) is the most common plasticizer used to increase the flexibility of polyvinyl chloride (PVC). DEHP is often used for the development of flexible plastics in food-packaging, plastic flooring, carpet material, roofing materials, plastic wall treatments, indoor decorations, wire, cable packaging materials, and children's toys [1]. DEHP is also used as a cleaner, industrial solvent, wetting agent, and lubricant [2]. Human exposure to DEHP usually occurs through air, water, or skin contact with DEHP-contained plastics [3]; however, the most common source of exposure is due to the ingestion of contaminated packaged foods. Due to its chemical structure, DEHP can readily dissolve and seep from the packaging materials into the food [2–4].

Phthalate exposure can have many potential health effects in humans. For example, a previous study reported that DEHP and mono-2-ethylhexyl phthalate (MEHP) can pass through the placenta and shorten the gestational period of a developing fetus [5]. DEHP also delays the development of the male reproductive system [2]. Recent evidences also show prenatal DEHP exposure is associated with shorter gestation [6], but prenatal DEHP exposure does not affect birth outcomes [7]. Even though oral ingestion of DEHP is one of the most common routes of exposure in humans, the effects of this toxin on gastric epithelial cells have not been fully elucidated.

The human gastric pathogen, *Helicobacter pylori* (*H. pylori*), is a spiral Gram-negative microaerophilic bacterium, which can selectively colonize the mucus layer of the stomach and can cause severe gastric problems including the development of chronic gastritis, peptic ulcers, and gastric cancer [8]. *H. pylori* is often transmitted to human through a variety of ways, including oral-oral and fecal-oral routes [9]. *H. pylori* infection induces apoptosis, of gastric epithelial cells, an effect which was reported with both in vivo [10, 11] and in vitro studies [12–14].

Plastics are widely used in food packaging in Taiwan and the world; however, DEHP exposure is often higher in the Taiwanese population than other countries such as in Germany or the US [15, 16]. *H. pylori* infection occurs in approximately 50% of the world's population. The Taiwanese population has as a 54.4% antibody seropositivity against *H. pylori* [17], which is higher than the *H. pylori* seropositivity in other countries, such as Ireland [18]. Taken together, these data demonstrate that the high exposure rate of DEHP and high infection rate of *H. pylori* in the Taiwanese people, as well as with the rest of the world, are likely an important health concern; however, the effect of DEHP and *H. pylori* coexposure on gastric epithelial cells is not well understood. For this reason, the effect of DEHP and *H. pylori* coexposure on gastric epithelial cell apoptosis, as an indicator of reduced epithelial cell integrity, was investigated in this study.

## 2. Materials and Methods

### 2.1. Cell Culture

A human gastric epithelial cell line, AGS (gastric adenocarcinoma. BCRC 60102), was purchased from the Cell Bank of the Taiwan National Health Research Institute and was grown in RPMI 1640 medium supplemented with 10% fetal bovine serum, 100 U/mL penicillin, and 100 *μ*g/mL streptomycin or 0.1% gentamicin (Hyclone, Logan, UT, USA). AGS cells were seeded at densities of 2~2.5 × 10^5^/35-mm culture dish or 5~5.5 × 10^5^/25 T flask, incubated for 24 hr, and switched to culture medium containing 0~80 *μ*M DEHP (CAS number: 117-81-7, Sigma-Aldrich, St. Louis, MO, USA). DEHP has limited solubility in water; a stock solution of DEHP was prepared in DMSO and subsequently diluted to various concentrations with cell culture medium. The final concentration of DMSO in culture medium was approximately 1% and had no significant effect on cell viability. All cultures were grown in a humidified incubator at 37°C and in an atmosphere of 95% air-5% CO_2_.

### 2.2. *H. pylori* Culture

Due to the role of cytotoxin associated gene A (CagA) and vacuolating cytotoxin A (VacA) genes, genes commonly associated with *H. pylori* associated gastric cell apoptosis [19], the CagA/VacA positive *H. pylori* strain ATCC 43054, purchased from the Cell Bank of the Taiwan National Health Research Institute, was used for this study. ATCC 43054 was cultured on trypticase soy agar with 5% sheep's blood (Curtin Matheson, Jessup, MD, USA) with Skirrow's selective antibiotic supplement (Prolab Inc., Scarborough, Canada) at 37°C in a CO_2_/O_2_ water jacketed incubator (Forma Scientific, Marietta, OH, USA) under microaerophilic conditions (10% CO_2_, 7.5% O_2_, 82.5% N_2_). *H. pylori* were added to cells at a bacterium: cellular concentration range (Multiplicities of infection, MOI) of 100 : 1 to 25 : 1. *H. pylori* was used between passages 5 and 15 for these experiments to ensure that the bacteria were able to readily adhere to AGS cells. Adherence was visualized using microscopy.

### 2.3. Cell Viability Assay

AGS cells were seeded in 48-well culture plates at a density of 100 cells/mm^2^, allowed to grow for 24 hr, and switched to culture media containing DEHP with or without *H. pylori* for 18 to 48 hr. For measuring the cell viability, MTT (3-[4,5-dimethylthiazol-2-yl]-2,5-diphenyltetrazolium bromide, 0.5 mg/mL; Sigma-Aldrich, St. Louis, MO, USA) was added to each well, and the plates were incubated for 3 hours at 37°C. The formazan crystals (a product of metabolic activity) were dissolved in isopropyl alcohol with 0.04% HCl and the formazan in each well was quantified using a Dynex MRX II spectrophotometer (Dynex Technologies, Chantilly, VA, USA) at absorption frequencies of 540 and 630 nm. The data were pooled from three independent experiments at least, and the number of replicate wells is ≥4.

### 2.4. DNA Fragmentation Assay

The extent of DNA fragmentation was quantified using the Cell Death Detection ELISA^Plus^ kit (Roche, Mannheim, Germany) as described in the manufacturer's manual. Briefly, cells were lysed by adding lysis buffer to each well and incubating for 30 min at 4°C. Each plate was centrifuged at 200 ×g for 10 min, and 20 *μ*L of each supernatant was transferred to streptavidin-coated wells. The wells were treated with an anti-histone- and anti-DNA-containing immuno-reagent, incubated for 2 hr at room temperature, washed three times, and treated with the peroxidase substrate 2,2′-azino-di-(3-ethyl-benzthiazoline sulfonate). Absorption at 405 nm was measured using a Dynex MRX II spectrophotometer (Dynex Technologies, Billingshurst, UK).

### 2.5. Immunoblotting Analysis of Caspase-3, Caspase-8, Bax, and Bcl-2

Cell protein extracts (20 *μ*g) were denatured in sodium dodecyl sulfate (SDS) sample buffer at 95°C for 5 min, loaded onto 10–20% gradient SDS-polyacrylamide gel electrophoresis (PAGE) gels (Invitrogen), and separated by electrophoresis. Separated proteins were transferred to a polyvinylidene difluoride (PVDF) membrane. The membrane was blocked with 5% nonfat milk in Tris-buffered saline (TBS) containing 0.05% Tween-20 and treated with antibodies against active caspase-3 (rabbit polyclonal antiactive caspase-3 antibody, 1 : 500, Abcam, Cambridge, MA, USA), active caspase-8 (rabbit polyclonal antiactive caspase-8 antibody, 1 : 1000, Abcam,), Bax (mouse polyclonal anti-Bax antibody, 1 : 500, Santa Cruz Biotechnology (SCBT), Santa Cruz, CA, USA), Bcl-2 (mouse polyclonal anti-Bcl-2 antibody, 1 : 500, SCBT), GAPDH (chicken polyclonal anti-GAPDH antibody, 1 : 1000, Millipore, Billerica, MA, USA) and *β*-actin (mouse monoclonal anti-*β*-actin antibody, 1 : 1000, SCBT). Bound antibodies were detected using HRP-conjugated secondary antibodies (1 : 5000, Jackson ImmunoResearch Laboratories; West Grove, PA, USA) and enhanced chemiluminescence (ECL, Amersham Pharmacia Biotech, Piscataway, NJ, USA). Densitometric images of blots were visualized on a BioSpectrum AC Imaging System (UVP, Upland, CA, USA) and were analyzed using Vision WorksLS analysis software.

### 2.6. Statistical Analysis

At least triplicate experiments were performed for each set of operating conditions. The quantitative data are expressed as mean ±SD Statistics were performed using SigmaStat 3.5 (SysStat Software, San Jose, CA, USA). Differences between study and control groups were evaluated by analysis of variance (ANOVA). The level of significance for differences between groups was further analyzed using post hoc Fisher's least significant difference (LSD) tests. A *P* < 0.05 was defined as statistically  significant.

## 3. Result

### 3.1. DEHP and *H. pylori* Coexposure Reduced Cell Viability of AGS Cells

**Figure 1:**
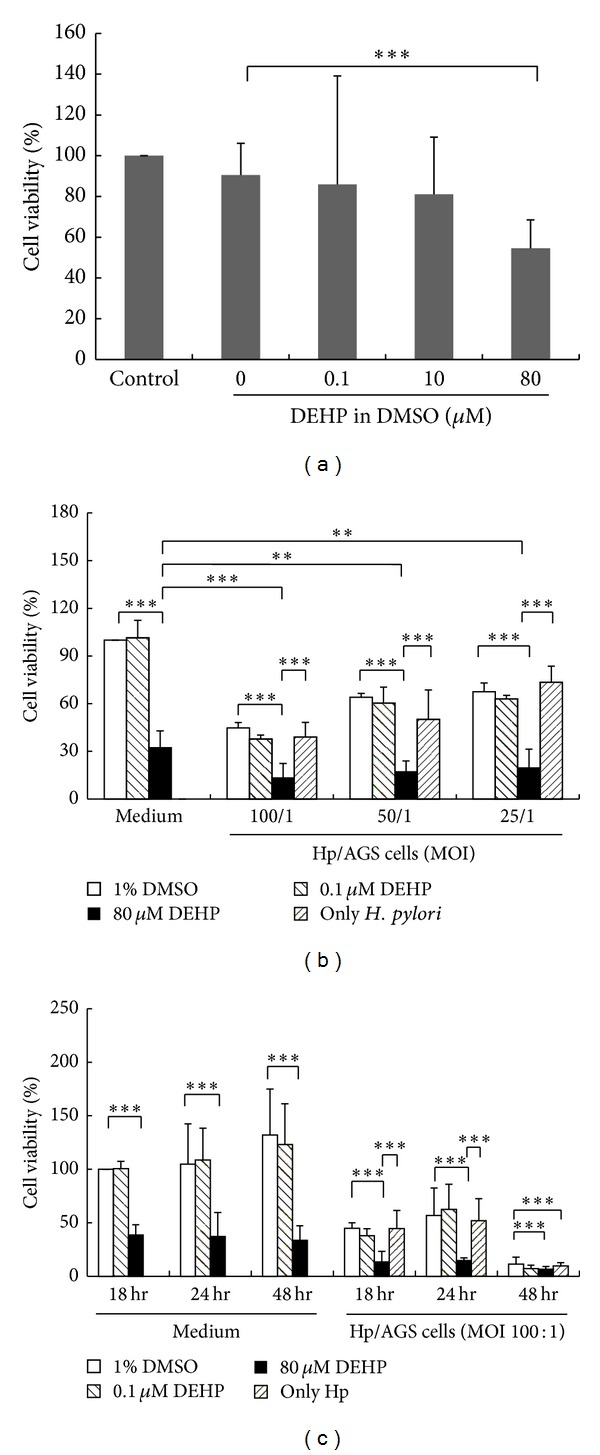
The cell viability of AGS cell. (a) Cells were treated with 0.1 to 80 *μ*M DEHP for 24 hours. (b) Cells were treated 0.1 and 80 *μ*M DEHP alone or combined with *H. pylori* (MOI = 100 : 1~25 : 1) for 18 hours (ten independent experiments). (c) Cells were treated 0.1 and 80 *μ*M DEHP alone or combined with *H. pylori* (MOI = 100 : 1) for 18, 24, and 48 hours Values obtained from MTT assay in mean + SD and normalized to nontreated value (*N* = 7, three independent experiments, **P < 0.05, ***P < 0.01, one way ANOVA assay with LSD *post hoc* test).

The cell viability of AGS cells after DEHP treatment for 24 hr is shown in Figure 1(a). Cell viability was significantly decreased after an exposure of 80 *μ*M of DEHP treatment (one way ANOVA, *P* < 0.001). The 0.1 *μ*M (*P* = 0.486), 1 *μ*M (*P* = 0.215), and 10 *μ*M (*P* = 0.375) of DEHP also induced a nonsignificant decrease in AGS cell viability, when compared with a DMSO (vehicle) group and tested by one way ANOVA. Additionally, no significant statistical difference (one way ANOVA, *P* = 0.427) was observed between control (culture medium without DMSO) and vehicle group. Since the DEHP-80 *μ*M caused the most significant decrease in cell viability, this concentration was utilized for further experiments.

AGS cell viability after DEHP and *H. pylori* coexposure are presented in Figure 1(b). Cell viability was significantly reduced by DEHP-80 *μ*M for 18 hr (one way ANOVA, *P* < 0.01). *H. pylori* infection significantly increased AGS cell death in a (multiplicity of infection) MOI-dependent manner. Compared with vehicle or *H. pylori* alone, DEHP-80 *μ*M and *H. pylori* coexposure significantly reduced the cell viability of AGS cell after an 18 hr exposure (one way ANOVA, *P* < 0.01). Compared with DEHP-80 *μ*M alone, DEHP-80 *μ*M and *H. pylori* coexposure also significantly reduced the cell viability of AGS cell after *H. pylori* (MOI = 100/1) coexposure (one way ANOVA, *P* < 0.01). Not only does this result imply a cytotoxic effect of DEHP and *H. pylori* alone on AGS cells, but it also indicates that the combined DEHP and *H. pylori* exposure has an additive cytotoxic effect on AGS cells. Since the cytotoxicity of *H. pylori* at a MOI of 100/1 when combined with DEHP was higher than at MOI of 50/1 and MOI of 25/1, this experimental condition was used for further studies. In addition, there was no significantly statistical difference in cell viability between the groups of *H. pylori* in DMSO (vehicle) and *H. pylori* (MOI = 100 : 1, 50 : 1 and 25 : 1) in culture medium (one way ANOVA, *P* = 0.613, *P* = 0.149, and *P* = 0.452 resp.).

A time-course study of AGS cell viability changes after DEHP and *H. pylori* coexposure are shown in Figure 1(c). The cell viability was significantly decreased in a time-dependent manner by DEHP-80 *μ*M treatment (one way ANOVA, *P* < 0.01). DEHP-80 *μ*M and *H. pylori* (MOI = 100 : 1) coexposure more significantly decreased AGS cell viability. Because almost cells (about 87%) were dead after 18 hr of exposure and cell viability was not significantly statistical difference between 18 hr and 24 hr after DEHP-80 *μ*M and *H. pylori* coexposure (one way ANOVA, *P* = 0.674), therefore, DEHP and *H. pylori* treatment for 18 hr was selected for further experiments.

### 3.2. DEHP and *H. pylori* Coexposure-Induced Apoptosis of AGS Cells

**Figure 2:**
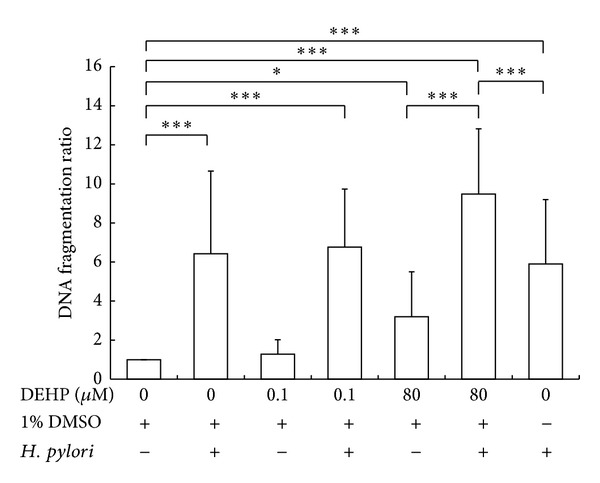
DEHP and *H. pylori* (MOI = 100 : 1) exposure induced DNA fragmentation of AGS cells. The level of apoptosis occurring with each treatment was determined by cell death ELISA^Plus^ kit. Statistical significance was analyzed by one-way ANOVA and post hoc LSD test (*P < 0.1, **P < 0.05, ***P < 0.01, *N* = 4).

The Cell Death Detection^Plus^ system (Roche) was used to detect AGS cell apoptosis (Figure 2). DEHP dose dependently induced a nonsignificant increase in the ratio of DNA fragmentation. Compared with a vehicle group, an 18 hr exposure of DEHP-80 *μ*M exhibited a nonsignificant tendency to increase the DNA fragmentation ratio (one-way ANOVA, *P* = 0.059). DEHP and *H. pylori* (MOI of 100/1) coexposure for 18 hr, however, significantly increased the DNA fragmentation ratio (one-way ANOVA, *P* < 0.01). The ratio of DNA fragmentation after DEHP-80 *μ*M and *H. pylori* coexposure was significantly higher than DEHP (one-way ANOVA, *P* < 0.01) or *H. pylori* alone (one-way ANOVA, *P* < 0.01). This result implies that DEHP can enhance the toxicity of *H. pylori* and increase *H. pylori*-*induced* apoptosis of AGS cells. Additionally, the ratio of *H. pylori*-induced DNA fragmentation did not exhibit a significant difference in DMSO-containing or normal medium, which indicates the activity and toxicity of *H. pylori* were not affected by DMSO.

### 3.3. DEHP and *H. pylori* Coexposure Increased Bax/Bcl-2 Ratio

**Figure 3:**
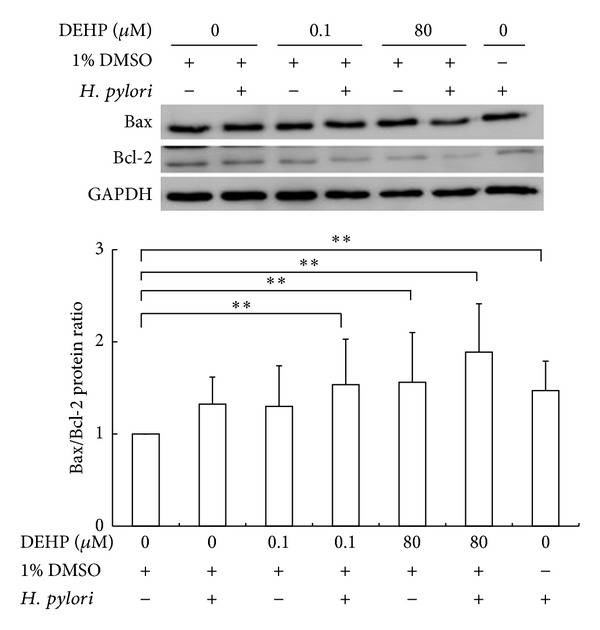
DEHP and *H. pylori* exposure for 3 hours changed the Bax/Bcl-2 ratio of AGS cells. Statistical significance was analyzed by one-way ANOVA and post hoc LSD test (**P < 0.05, *N* = 4).

Expression of Bax and Bcl-2 protein after DEHP and *H. pylori* treatment for 3 hours was detected by Western blot analysis and the Bax/Bcl-2 ratio was calculated. DEHP-80 *μ*M (one way ANOVA, *P* < 0.05) or *H. pylori* treatment alone (one way ANOVA, *P* < 0.05) increase Bax/Bcl-2 ratio; however, the combined treatment of DEHP-80 *μ*M and *H. pylori* coexposure caused a much higher Bax/Bcl-2 ratio increase (one way ANOVA, *P* < 0.05) (Figure 3). This result implied that DEHP and *H. pylori* coexposure disturbed the balance between Bax and Bcl-2 protein expression.

### 3.4. Effect of DEHP and *H. pylori* Coexposure on Expression of the Active Forms of Caspase-3 and Caspase-8

**Figure 4:**
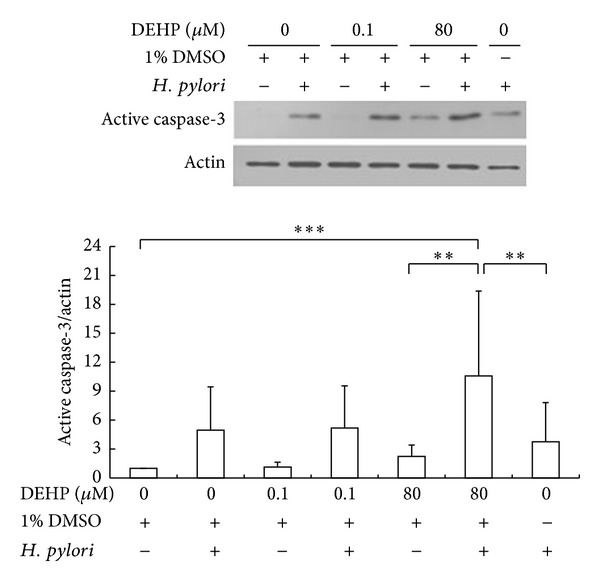
DEHP and *H. pylori* increased active form of caspase-3 protein expression after 18-hour treatment. Statistical significance was analyzed by one-way ANOVA and post hoc LSD test (**P < 0.05, ***P < 0.01, *N* = 4).

**Figure 5:**
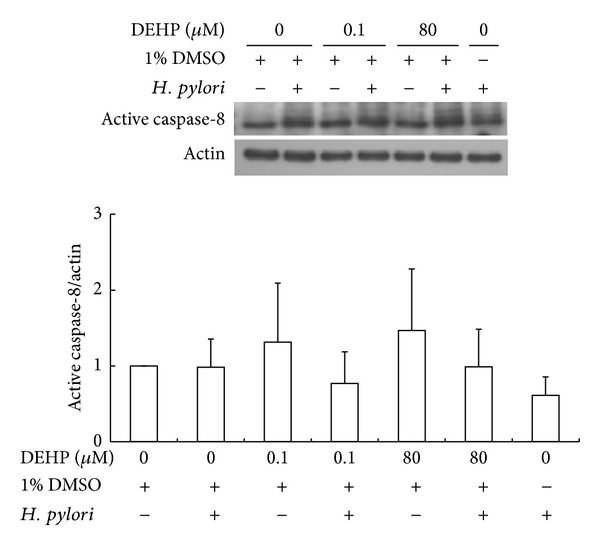
The effect of DEHP and *H. pylori* on active caspase-8 protein expression after 18-hour treatment. Statistical significance was analyzed by one-way ANOVA and post hoc LSD test (*N* = 3).

Expression of active caspase-3 and caspase-8 protein after 18 hr DEHP and *H. pylori* treatment was quantified by Western blot analysis and the results are presented in Figures 4 and 5. Expression of active caspase-3 was significantly increased after *H. pylori*/DEHP co-exposure, especially with DEHP-80 *μ*M combined with *H. pylori* treatment (one way ANOVA, *P* < 0.01, compared with vehicle group) (Figure 4). DEHP-80 *μ*M/*H. pylori* co-exposure-induced active caspase-3 protein expression, which was also higher than DEHP-80 *μ*M (one way ANOVA, *P* < 0.05) or *H. pylori* alone (one way ANOVA, *P* < 0.05). Active caspase-8 expression was not significantly changed after 18 hr DEHP or *H. pylori* (one way ANOVA, *P* > 0.1) (Figure 5). The ratio of these apoptosis-related proteins also was summarized in Table 1 with mean ± SD.

## 4. Discussion

*Helicobacter pylori* (*H. pylori*) bacterium was first identified and isolated from gastric biopsies of patients with gastritis and peptic ulcers [20]. The NIH Consensus Development Conference [21] identified *H. pylori* as the primary reason for peptic ulcer development. *H. pylori* was also evaluated by the International Agency for Research on Cancer (IARC) and was identified as a human Group 1 carcinogen (1994). *H. pylori* is associated with both gastric adenocarcinoma and gastric lymphomas. *H. pylori* is transmitted to humans through a variety of ways, including oral-oral and fecal-oral routes [9].

DEHP is a plasticizer found widely in food packaging, which often can migrate from the plastic wrapping and actually contaminate the packaged food [22]. DEHP contamination is prevalent throughout the food chain [23]. Meat, fish, dairy products, fresh fruit, and bread all exhibited 100% prevalence for DEHP contamination. Other important foods also exhibited a prevalence for DEHP contamination to a lesser extent; cereals and legumes (93%), vegetables (80%), and condiments (66%) [24]. The estimated daily intake of phthalates in the general Taiwanese population is approximately 0.1 to 309.6 *μ*g/kgBW/day (about 0.015 to 47.5 *μ*M by a 60 kg adult) [15]. With PVC industry workers, the daily intake of phthalates is much higher at about 0.6–850 *μ*g/kgBW/day (about 0.092 to 130.6 *μ*M by a 60 kg adult) [25]. Since the half-life of DEHP in the human body ranges between 16 and 24 hours [26], DEHP may be able to stimulate the epithelial cells of the gastrointestinal tract and accumulate in the human body via sustainably ingested of DEHP-contaminated foods. In humans, ingestion of 10 grams of DEHP (~426 *μ*M, assuming 60 kg b.w.) can cause mild gastric disturbances and “moderate catharsis” [27]; however, the effects of short and long term high level exposure to DEHP are not known. In this study, the effects of *H. pylori* and DEHP were identified on gastric epithelial cells. We found that DEHP and *H. pylori* coexposure decreased AGS cell viability greater than DEHP or *H. pylori* alone (Figure 1), indicating that the combined exposure of *H. pylori* and DEHP has additive toxic effects on gastrointestinal epithelial cells, potentially altering the ratio between cell proliferation and apoptosis.

The imbalance between cell proliferation and apoptosis may contribute to gastric carcinogenesis. Gastric resection specimens from patients that exhibited normal gastric mucosa contained a low number of apoptotic cells at the surface epithelium. The apoptotic number was significantly increased in cases with chronic gastritis and/or intestinal metaplasia [28]. Increased apoptosis is associated with the development of gastric carcinoma [29]. The TUNEL assay on biopsies obtained from the gastric mucosa in patient with gastric carcinoma also found multiple apoptotic cells [30]. In addition, it has been shown that the increase rate of *H. pylori*-*induced* apoptosis on AGS cell line was 47.0% and on a normal gastric epithelial cell line (GES-1), which is developed by Beijing Institute for Cancer Research Collection, was 113.0% [31], the flow cytometry experiment also showed the ratio of apoptosis in GES-1 cells was 10.2%~27.6% after *H. pylori* infection for 4 hours [32], and the ratio of apoptosis in AGS cells was about 18% after *H. pylori* infection for 6 hours [33]. These articles indicated that normal gastric epithelial cell might be more sensitive than AGS cell to *H. pylori* and could cause higher ratio apoptosis than AGS cell after *H. pylori* infection. Because apoptosis ratio increase is one of the possible pathways which associated with the development of gastric carcinoma [29], *H. pylori*-*induced* carcinogenesis might be more evident on normal gastric epithelial cell than AGS cells. In this study, we found that *H. pylori* and DEHP coexposure increased DNA fragmentation of AGS cells (Figure 2). This result implies that *H. pylori* and DEHP coexposure might induce cell apoptosis and alter the balance between cell proliferation and cell death in gastric epithelial cells, disrupting the integrity of the gastric mucosa and promoting gastric carcinogenesis. Moreover, the ratio of *H. pylori* and DEHP coexposure was higher than *H. pylori* infection alone, further indicating that DEHP enhances *H. pylori*-*induced* apoptosis on AGS cells. Apoptosis has been shown that, related with the cell subpopulations of highly growth rate selection in gastric precancerous lesions and involved in the malignant transformation [34], *H. pylori* is type I carcinogen and DEHP is type 2B carcinogen in IARC classification (2000); moreover, we found DEHP and *H. pylori* exposure increased AGS cell apoptosis. Therefore, DEHP and *H. pylori*-*induced* AGS cell apoptosis might increase the AGS cell malignant transformation.

Previous articles also revealed that apoptosis-related proteins expression was different in various types of gastric precancerous lesions and might involve in the process of carcinogenesis and metastasis. Bcl-2 protein expression was increased in gastric premalignant lesions and decreased its expression after malignant change [34, 35]. Bax protein expression was upregulated in patient's gastric precancerous lesions after *H. pylori* infection [36]. Bax protein is also highly expressed in intestinal metaplasia regions nearby to tumors and related with induction of apoptosis [35]. Bax protein was found highly expression in gastric cancer patient's tissues and there was no difference in the tumor stage [37]. Taken together, the Bax/Bcl-2 ratio of cancer cell might increase after malignant change. Our results show that combined exposure of DEHP and *H. pylori* increased the Bax/Bcl-2 ratio of AGS cell (Figure 3), this result might imply DEHP, and *H. pylori* exposure might induce AGS cell malignant change.

Apoptosis includes two separate pathways: intrinsic and extrinsic. The intrinsic pathway is induced by the permeability loss of the mitochondrial outer membrane. The permeability loss of the mitochondrial outer membrane leads to cytochrome c release, apoptosome formation, and procaspase-9 activation. The extrinsic pathway is initiated by extracellular death ligand binding, which results in the activation of caspase-8. Both the intrinsic and extrinsic pathways activate caspase-3 and result in apoptosis [38, 39]. *H. pylori* was reported to induce gastric epithelial cell apoptosis through the activation of the extrinsic pathway [40]; however, in a different study, *H. pylori* was also reported to induce apoptosis mainly through the intrinsic pathway. Caspase-8 activation did not appear to play a major role in *H. pylori* induced apoptosis [41]. In this study, the DEHP and *H. pylori* coexposure significantly increased activated caspase-3 in AGS cells (Figure 4), but caspase-8 activation was not changed after an 18 hr exposure (Figure 5). This result is similar to previously reviewed articles, indicating that caspase-8 is not essential for DEHP or *H. pylori*-*induced* apoptosis and that DEHP and *H. pylori*-*induced* apoptosis is also likely mediated by the intrinsic pathway. Additionally, active caspase-3 was significantly higher in cells that were co-exposed to DEHP and *H. pylori* than cells exposed to DEHP or *H. pylori* alone. These results paralleled the DNA fragmentation study, indicating DEHP and *H. pylori* coexposure has an additive effect on caspase-3 activation and apoptosis of AGS cells. Moreover, caspase-3 activation plays an important role in stress-induced invasion [42], high level caspase-3 expression in the tissue sample of gastric cancer patients has been shown poor prognosis and related with gastric cancer lymph node metastasis [43]. The caspase-3 protein expression in primary gastric carcinoma was higher than metastatic gastric carcinomas [44, 45]. This study found that DEHP and *H. pylori* exposure increased the expression of active form of caspase-3 in AGS cells and induced AGS cells apoptosis, and this result implied that DEHP and *H. pylori* might enhance the ability of stress-induced invasion ability of gastric cancer. Taken together, these data not only indicate that while caspase-3 likely plays an important role in AGS cell apoptosis induced by the combined DEHP and *H. pylori* exposure, but also imply the additive effect on malignant transformation of AGS cell after DEHP and *H. pylori* exposure.

In conclusion, this paper reports that DEHP and *H. pylori* coexposure can regulate Bax and Bcl-2 protein expression to increase Bax/Bcl-2 ratio, activate caspase-3 protein, and enhance AGS cellular apoptosis. These results provide new information about the carcinogenetic effect of DEHP and *H. pylori* coexposure on gastric epithelial cells, which may, with further research, suggest a possible mechanism, in which DEHP enhances carcinogenesis when combined with a known carcinogen. Therefore, a further investigation is necessary to understand the underlying mechanism by which DEHP and *H. pylori* can induce carcinogenesis and metastasis in gastric epithelial cells.

## Figures and Tables

**Table 1:** Summary of apoptosis-related proteins expression ratio after DEHP alone or DEHP combined with* H*. *pylori* (MOI = 100 : 1) treatment^a^.

Ratio	*N*		Medium (1% DMSO)		Medium (1% DMSO) + Hp/AGS cells (MOI 100 : 1)		Medium (w/o DMSO) + Hp/AGS cells (MOI 100 : 1)	
			Mean	SD	Mean	SD	Mean	SD
Active caspase-3/actin	4	Vehicle	1.00	0.00	4.95	4.49		
		0.1 uM DEHP	1.14	0.50	5.17	4.36		
		80 uM DEHP	2.23	1.17	10.57	8.81		
		Only *H*. *pylori *					3.75	4.05

Active caspase-8/actin	3	Vehicle	1.00	0.00	0.98	0.37		
		0.1 uM DEHP	1.31	0.78	0.77	0.42		
		80 uM DEHP	1.47	0.81	0.99	0.50		
		Only *H*. *pylori *					0.61	0.24

Bax/Bcl-2	5	Vehicle	1.00	0.00	1.33	0.29		
		0.1 uM DEHP	1.30	0.44	1.54	0.49		
		80 uM DEHP	1.56	0.54	1.89	0.53		
		Only *H*. *pylori *					1.47	0.32

^a^The protein expression ratio was normalized to vehicle group.
